# 850 Reconstruction of Extensive Burn Defects with Multiple Flaps: A Case Series

**DOI:** 10.1093/jbcr/iraf019.381

**Published:** 2025-04-01

**Authors:** Mare Kaulakis, Hilary Liu, José Arellano, Christopher Fedor, Francesco Egro

**Affiliations:** University of Pittsburgh School of Medicine; University of Pittsburgh Medical Center; University of Pittsburgh Medical Center; University of Pittsburgh School of Medicine; University of Pittsburgh Medical Center

## Abstract

**Introduction:**

Full-thickness burns present a significant reconstructive challenge due to the extensive tissue damage they inflict, often extending through skin and underlying tissues to deep muscle and bones. Traditional reconstructive techniques such as skin grafting may not provide adequate coverage and vascularity for optimal functional and aesthetic recovery. This study aims to investigate the effectiveness of utilizing multiple flaps to reconstruct a single anatomical region, offering an innovative approach in the treatment of severe burn injuries.

**Methods:**

A retrospective review of all patients who underwent multi-flap reconstruction of extensive burn defects at a single ABA-verified burn center from 2010 to 2023 was conducted. Collected data included demographic information, burn characteristics, type of flaps, and complications.

**Results:**

Six male patients (mean age of 49 ± 19.52 years) requiring multi-flap intervention for seven burn defects were included in the study. Average BMI was 27.82 ± 7.80, with common comorbidities including diabetes (50%; n=3) and smoking (16.67%; n=1). All patients suffered from full-thickness thermal burns with an average total body surface area (%TBSA) of 40.64 ± 21.53.

Multi-flap operation was indicated due to bone exposure of the lower extremity in five patients (4 gastrocnemius and soleus muscle flaps, 1 bilateral lateral and medial gastrocnemius flap following above-knee amputation). One patient received an extensor carpi radialis and flexor carpi ulnaris muscle flap for acute coverage of an upper extremity burn.

Four patients had wound healing complications, three of which required additional interventions comprising of excision plus the application of a split-thickness skin graft or skin substitute. One patient had a partial flap loss that had been previously anticipated due to potential unviability of the muscle, and one patient had sepsis. No hematoma, seroma, venous or arterial thrombosis were reported. Ultimately, successful limb salvage was achieved in three patients and coverage of amputation defects in one patient. Two patients died from respiratory failure a month after trauma admission.

**Conclusions:**

This novel technique of combining multiple free flaps for coverage of large burn deformities offers a good solution to cover large area deficits. This technique should be considered for extensive burn injuries due to its favorable outcomes.

**Applicability of Research to Practice:**

Employing multiple flaps for coverage of large burn deformities can significantly enhance both functional and aesthetic outcomes. This study’s successful findings advocate for the incorporation of multiple flaps into clinical practice, providing a foundation for further research to standardize multi-flap reconstructive approaches in severe burn care.

**Funding for the Study:**

N/A

